# COVID-19 Outbreak: Understanding Moral-Distress Experiences Faced by Healthcare Workers in British Columbia, Canada

**DOI:** 10.3390/ijerph19159701

**Published:** 2022-08-06

**Authors:** Esther Alonso-Prieto, Holly Longstaff, Agnes Black, Alice K. Virani

**Affiliations:** 1 BC Women’s Hospital and Health Centre, 4500 Oak St., Vancouver, BC V6H 3N1, Canada; 2 Faculty of Medicine, University of British Columbia, 2329 West Mall, Vancouver, BC V6T 1Z4, Canada; 3 Provincial Health Services Authority, 1333 W Broadway, Vancouver, BC V6H 4C1, Canada; 4 Faculty of Health Sciences, Simon Fraser University, 8888 University Drive, Burnaby, BC V5A 1S6, Canada; 5 Providence Health Care, 1081 Burrard St., Vancouver, BC V6Z 1Y6, Canada; 6 Department of Medical Genetics, University of British Columbia, Vancouver, BC V6T 1Z4, Canada

**Keywords:** COVID-19, moral distress, ethical dilemmas

## Abstract

Pandemic-management plans shift the care model from patient-centred to public-centred and increase the risk of healthcare workers (HCWs) experiencing moral distress (MD). This study aimed to understand HCWs’ MD experiences during the COVID-19 pandemic and to identify HCWs’ preferred coping strategies. Based on a qualitative research methodology, three surveys were distributed at different stages of the pandemic response in British Columbia (BC), Canada. The thematic analysis of the data revealed common MD themes: concerns about ability to serve patients and about the risks intrinsic to the pandemic. Additionally, it revealed that COVID-19 fatigue and collateral impact of COVID-19 were important ethical challenges faced by the HCWs who completed the surveys. These experiences caused stress, anxiety, increased/decreased empathy, sleep disturbances, and feelings of helplessness. Respondents identified self-care and support provided by colleagues, family members, or friends as their main MD coping mechanisms. To a lesser extent, they also used formal sources of support provided by their employer and identified additional strategies they would like their employers to implement (e.g., improved access to mental health and wellness resources). These results may help inform pandemic policies for the future.

## 1. Introduction

As with other healthcare systems across the world, the early months of 2020 saw the beginning of the COVID-19 pandemic and its effect across the Canadian healthcare system. The impact was widespread and included not only an increase in hospitalizations of patients with the virus but also other ripple effects, including scarcities of supplies such as Personal Protective Equipment (PPE) and staffing shortages. Additionally, throughout the pandemic, healthcare providers in British Columbia (BC) were faced with balancing professional duties and responsibilities with personal considerations including public backlash [1] and their own health issues, which could include the effects of long COVID. In many cases, healthcare workers were redeployed due to staff becoming sick or needing to isolate. Numerous surgeries and healthcare visits were postponed, and there was a shift toward virtual visits and/or decreased visits overall.

At this time, many morally complex issues were encountered throughout the healthcare system, including ethically fraught decisions related to resource allocation, duty to care, vaccine prioritization, exacerbations of social and systemic inequities, and being required to make choices when data were suboptimal [2,3,4].

Evidence from previous pandemics such as the 2003 severe acute respiratory syndrome (SARS) outbreak, the 2009 H1N1 influenza outbreak, and the 2014 Ebola outbreak indicates that having to face morally complex situations has a strong impact on healthcare workers’ emotional well-being and can lead to increased moral distress and injury [5,6,7,8,9,10]. Moral distress occurs when an individual identifies the ethically appropriate action, but that action conflicts with personal values, perceived obligations, or institutional constraints [11,12]. When moral distress is severe and is left unresolved, it may lead to moral injury [13].

Recent studies have shown that, as with previous public health emergencies, the COVID-19 pandemic has also led to moral distress experiences among nurses [14,15,16,17,18], physicians [15,16,17,18,19,20], and non-clinical healthcare workers [17,18]. Additionally, it has been suggested that COVID-19-driven moral injury remains stable for three months, even while moral distress declines [19]. However, the cause and nature of the moral distress related to the COVID-19 pandemic requires further exploration, to determine how this experience manifests across different geographical regions and stages of the pandemic response.

This study is unique in that it sought to gain a better understanding and broader view of the moral distress experiences of BC healthcare workers (HCWs) during the COVID-19 pandemic. To achieve this goal, participants from varied professional backgrounds were invited to complete online surveys over different stages of the pandemic response. The ultimate aim of the project was to identify effective ways to enhance individual and organizational resilience, in order to support the healthcare system in managing pressures related not only to pandemics but also to other unknown or known potential stressors on the healthcare system such as climate change events and aging populations.

## 2. Methods

### 2.1. Study Design

In designing the study, we relied on interpretive description methodology, an established approach to qualitative-knowledge development within the applied clinical fields, proposed by Thorne and colleagues [21,22,23]. Interpretive description supports the process of describing and interpreting the lived world as experienced in everyday situations to capture themes and patterns. Therefore, its goal is not to study a representative sample to allow for generalizing findings to a wider population but to explore, describe, and explain human experience.

The study design was also informed by two contextual characteristics of the COVID-19 outbreak: (1) the existence of significant differences in terms of impact, infection rates (i.e., ‘waves’), and management across geographical regions; and (2) the unprecedented dynamic nature and scale of the impact on society. Thus, survey questions were original and unvalidated, developed based on qualitative feedback from the population under study instead of using established moral-distress surveys, which were developed and validated under different circumstances and with different populations. In the first survey, respondents answered a series of open-ended questions, and the results were analysed for common themes. The most common themes were then used to construct the second and third surveys, which were deployed to validate and assess changes in the expressed themes over time.

Ethics approval to conduct this study was obtained from the University of British Columbia’s Behavioural Ethics Board (H20-01104).

### 2.2. Participant Recruitment

We used purposive sampling, a strategy commonly employed in qualitative research, to identify information-rich cases [24]. The study was restricted to one Canadian province, BC. Individuals were eligible to participate if they were employed by one of the six provincial healthcare authorities that provided clinical care (including in-patient care, long-term care, pre-hospital care, and out-patient clinics) during the COVID-19 pandemic. There were no specific exclusion criteria other than working in a health authority, as we wished to capture experiences of moral distress at all levels of the healthcare system. To identify participants, study team members disseminated invitation letters through list-serves, posters, and presentations and by snowball sampling.

### 2.3. Data Collection

**Figure 1:**
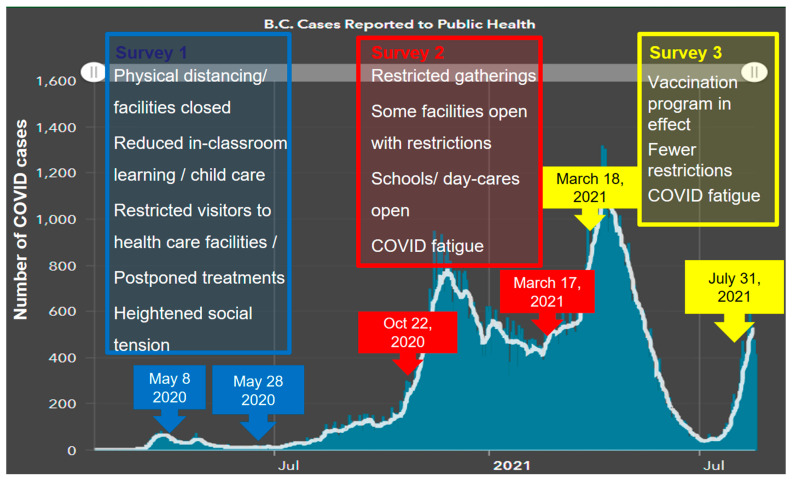
Timeline of surveys superimposed on a graph depicting the waves of COVID-19 cases in BC, Canada as published by BC Centre for Disease Control (https://experience.arcgis.com/experience/a6f23959a8b14bfa989e3cda29297ded, accessed on 20 June 2022). Most relevant public-health measures in effect during each survey period are also summarized [25].

Three surveys were distributed to BC healthcare employees between May 2020 and July 2021 (see Supplementary S1 and S2). All surveys included demographic questions such as age, gender, health authority, religious affiliation, ethnicity, role, number of years in the role, and area of service and questions related to moral distress. The questions related to moral distress varied between surveys, as they were adjusted to align with different stages of the provincial response to the pandemic (Figure 1) and to probe themes arising in the concurrent analysis. It is worth noting that, while the vast majority of healthcare workers were vaccinated for COVID-19 in early 2021, the vaccine order that mandated all BC HCWs to be vaccinated (or be put on unpaid leave) came into effect on 26 October 2021, after these surveys were completed.

Survey 1 was deployed between 8 May and 28 May 2020, at a time when the number of COVID-19 cases in BC had just started to increase, several restrictive measures were in place, and there was heightened social uncertainty that precipitated coping behaviours, such as panic buying (Figure 1). This survey included mostly open-ended questions that were then coded for common themes. The most common themes were used to develop the closed-ended questions for Surveys 2 and 3 to validate and assess changes in these findings over time. Free-text options were also included in Surveys 2 and 3 to allow participants to describe new experiences. Survey 2 was deployed between 22 October 2020 and 17 March 2021. During this period, there was a significant and stable increase in the number of COVID-19 cases, and gatherings were still restricted, but some facilities were able to operate to some extent, and schools and daycares were open (Figure 1). The third survey was distributed between 18 March 2021 and 31 July 2021, at a time when the number of COVID-19 cases reached the highest peak in BC and started to decrease again, the widespread public vaccination program was launched, and some restrictions, such as on small gatherings, were lifted (Figure 1).

### 2.4. Data Analysis

Sociodemographic data were analysed using descriptive statistics. Qualitative data analysis was conducted simultaneously with data collection, each informing the other in an iterative process. The analysis followed Braun and Clarke’s 6-step framework [26] to identify themes and patterns of meanings across the dataset. This method involves the following steps: (1) reading and familiarization, (2) coding, (3) generating themes, (4) reviewing themes, (5) defining and naming themes, and (6) finalizing the analysis [26].

## 3. Results

A total of 135 HCWs completed Survey 1, 320 completed Survey 2, and 145 completed Survey 3. As shown in Table 1, the majority self-reported as White females between the ages of 31 and 60.

Participants represented a diverse collection of professional backgrounds including nurses, physicians, paramedics, allied health professionals, researchers, administrative staff, managers, and executives.

**Figure 2:**
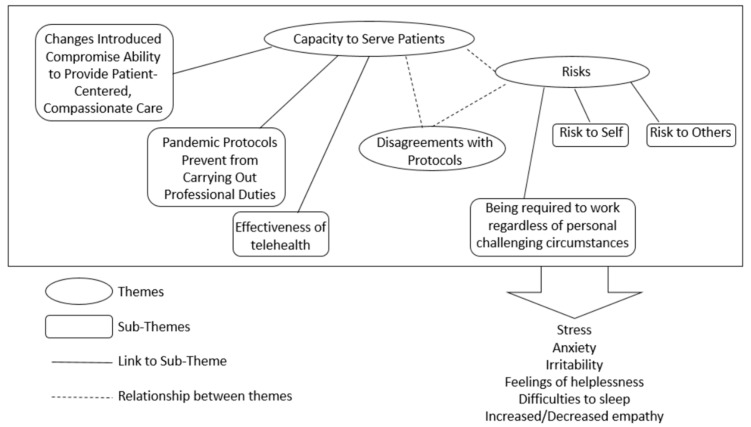
Graphical depiction of the thematic framework showing 3 main themes and 6 sub-themes underlying moral distress experiences of BC HCWs during COVID-19.

Most participants across all surveys stated that they were experiencing moral distress in their work (Survey 1 = 60%, Survey 2 = 69%, and Survey 3 = 68%). When asked to describe their experiences, several interrelated themes emerged from the open-ended responses of Survey 1 and continued to be expressed by respondents of Surveys 2 and 3 (Figure 2), as described in more detail in the next sections. 

### 3.1. Experiences of Moral Distress

#### 3.1.1. Theme 1: Healthcare Professionals’ Capacity to Serve Patients

As shown in Figure 2, the main theme emerging from Survey 1 centred on the HCWs’ capacity to serve patients. This theme included three sub-themes: changes introduced compromise the ability to provide patient-centred, compassionate care; pandemic protocols prevent HCWs from carrying out their professional duties; and the effectiveness of telehealth (Figure 2).

As explained by a Survey 1 participant: “*The very pillars of healthcare and social work practice: patient centered-care, consent to accept risks, right to self-determination/agency, are no longer upheld*”.

**Figure 3:**
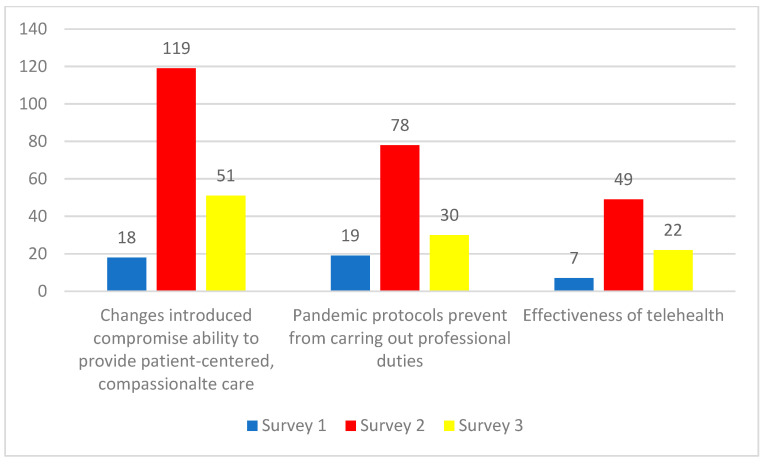
Number of responses corresponding to theme 1: HCWs’ capacity to serve patients. Since participants could select multiple themes, it is not possible to calculate percentages.

These themes were corroborated and further explicated by participants responding to Surveys 2 and 3 (Figure 3). For example, a Survey 2 participant wrote: *“I’ve witnessed a steep decrease in quality of care that can be provided by myself and other colleagues due to restrictive measures during an outbreak (…)”.* A Survey 3 participant similarly wrote: *“We see clients based on a waiting list; however, clients eligible for service were skipped over if they needed interpreters or had more complex needs which were difficult to meet under pandemic management protocols”.*

Some respondents across all three surveys expressed concerns about how the effectiveness of telehealth and how a shift towards virtual visits could impact their “capacity to serve patients” with certain populations (Figure 3). For example, a Survey 2 participant stated: *“I am very limited in my face-to-face encounters with my clients due to COVID (related) precautions and many of the ways I would have been able to support them are currently on hold. Almost none of my clients have phones/other means to tele-communicate so I really rely on face-to-face encounters”.*

#### 3.1.2. Theme 2: Risks

A second theme emerging during the initial stage of the pandemic response (Survey 1) centred on the risks that healthcare professionals were facing (Figure 2). One sub-theme focused on concerns over risks to themselves, while a second sub-theme focused on risks to colleagues, family members, and friends (Figure 2). For example, a Survey 1 participant wrote *“I had to express my concerns to senior leadership and refuse to participate in a plan that was putting [my colleagues] at risk”.*

**Figure 4:**
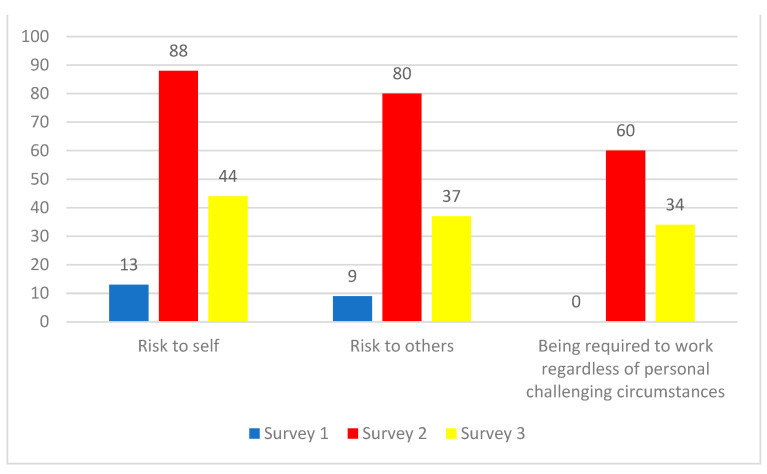
Number of responses corresponding to theme 2: Risks of COVID-19 infection and transmission. Since participants could select multiple themes, it is not possible to calculate percentages.

These sub-themes were corroborated by participants of Surveys 2 and 3 (Figure 4). Thus, one Survey 2 participant wrote “*I felt very scared about possibly having to provide direct clinical care, which I don’t usually do. My family has risks for severe COVID-19 and poor outcomes*”. While a Survey 3 participant wrote: “*I was asked by my manager to swap roles with a colleague in a high-risk area of the hospital early on during the pandemic due to their pre-existing health conditions. I felt uncomfortable doing so because little was known about how COVID-19 was transmitted. It felt unfair that I was asked to put myself at risk in place of another colleague*”.

One additional sub-theme related to the theme “Risk” also emerged from Surveys 2 and 3, which centred on participants being required to work regardless of personal challenging circumstances (Figure 2 and Figure 4). Thus, one Survey 3 participant wrote: *“Personal and family struggles related to COVID-19 stress has been difficult. I have felt forced to put my work over my family because it is so busy, and it will let my team and patients down if I call in sick to take care of my family’s mental health”.* Another participant wrote *“I have friends that work at the hospital who have immunocompromised spouses or roommates that were still required to work in high-risk areas (like emergency department) and management was not supportive of them temporarily moving to a lower risk area”.*

#### 3.1.3. Disagreements with the New COVID-19 Protocols

**Figure 5:**
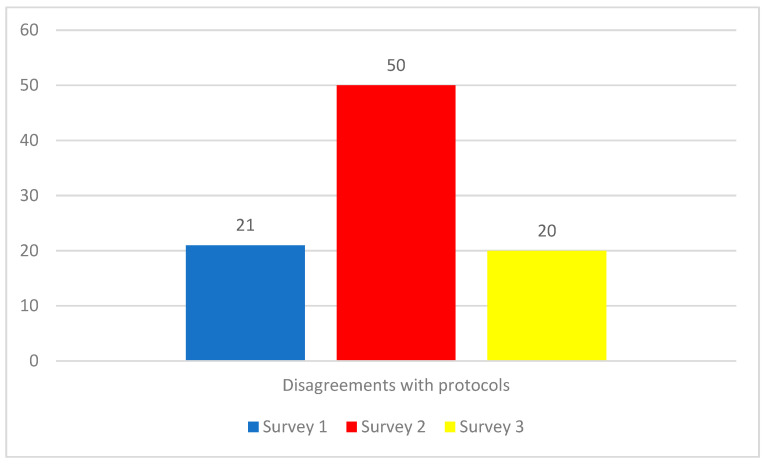
Number of responses corresponding to theme 3: Disagreement with COVID-19 protocols. Since participants could select multiple themes, it is not possible to calculate percentages.

A third theme was focused on disagreements with the new COVID-19 protocols (Figure 2 and Figure 5). Participants stated that their disagreements with the new protocols were causing moral distress. For example, a Survey 2 participant explained that *“It is not clear that there is a balance between the cost/benefit of some significant changes”.* Similarly, a Survey 3 participant was concerned that *“The restrictions were not always supported by logic or the current epidemiology”.* The reasons for the disagreements were diverse, including disagreements related to scientific understanding, operational concerns, or the provision of care.

### 3.2. Impact of Moral Distress

**Figure 6:**
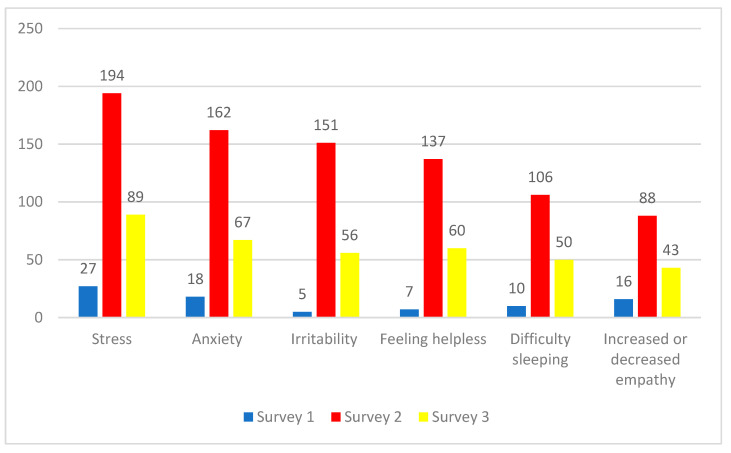
Number of responses on the impact of moral distress. Since participants could select multiple themes, it is not possible to calculate percentages.

The impact of moral distress on survey respondents is shown in Figure 6. By far the most common impacts were reports of stress, anxiety, and irritability. Many also expressed feelings of helplessness, had difficulty sleeping, and reported that their experience of moral distress had either increased or decreased their ability to empathize with others (both increased and decreased empathy were explained in a negative manner). For example, a Survey 2 participant expressed, *“I’m now having literal nightmares about lack of vaccine, lack of PPE etc., especially on the night before I come back to work”.* While a Survey 3 participant said that they were *“Suffering from PTSD and will need counseling. Unfortunately, there is no time to find help right now with the workloads, demands, and endless amounts of needed overtime. We are forced to decide whether we leave our colleagues working short or to put our mental health first. Always, mental health is pushed aside”.* While yet another Survey 3 participant said *“I am burnt out. I would like to leave the healthcare profession. At this point I don’t feel that the financial compensation is worth the mental and physical distress”.*

### 3.3. Current and Anticipated Ethical Challenges

Survey respondents were also asked to describe the main ethical challenges they believed HCWs currently faced or would be facing in response to the COVID-19 pandemic. Table 2 shows responses in descending order of popularity with more detailed explanations of each theme provided below.

#### 3.3.1. COVID-19 Fatigue

A new theme that arose in Surveys 2 and 3 was the most popular overall in response to the question about current or future ethical challenges. This theme referred to being tired of all COVID-19 related matters. As stated by a Survey 2 participant, “*I* *for sure have COVID fatigue. Definitely, I have compassion fatigue. I am snippy with my colleagues. I am exhausted helping family, patients, and my colleagues deal with their lives and issues. This has been a hard time and serious struggle”.*

#### 3.3.2. Collateral Impacts of COVID-19

A second new theme that emerged in Surveys 2 and 3 as an ethical challenge that respondents were facing or could face in the future referred to the collateral impacts of COVID-19, including exposing social inequities in healthcare and effects on the overall population’s mental health. For example, a Survey 2 participant wrote: *“The collateral impacts of the Covid restrictions and policies are a huge problem and it feels as if it’s not being talked about or acknowledged enough beyond the front-line. Many healthcare workers see it and worry about it every day and it’s extremely upsetting”.* While another participant wrote: “*I am concerned about the social inequities and further impact on families that are already challenged–reduced access to services, technology, mental health, safety”.*

#### 3.3.3. Additional Current or Anticipated Sources of Ethical Challenges

Other themes that were considered by the participants to be a current or future source of ethical challenges were similar to previous answers about moral-distress experiences. These themes were again centred around the ability to serve patients, disagreements with the implemented pandemic management protocols and the risks faced by the participants, their colleagues, or family members, and to a lesser extent the effectiveness of telehealth.

Interestingly, participants stated that not having a safe environment to discuss disagreements with colleagues or leadership regarding the COVID-19 protocols that should be implemented was also identified as a current or future ethical challenge.

### 3.4. Sources of Support

**Figure 7:**
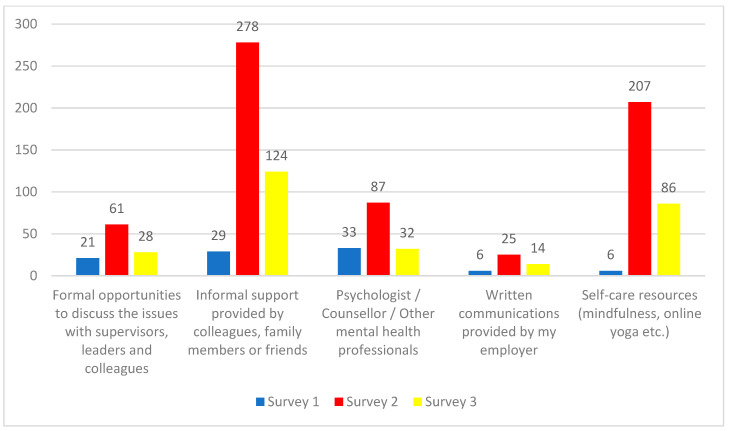
Sources of support to cope with negative psychological impacts of COVID-19. Since participants could select multiple themes, it is not possible to calculate percentages.

Respondents were also asked to identify the main sources of support they had used to cope with the negative psychological impacts of COVID-19. Informal resources such as self-care resources and support provided by colleagues, family members, or friends were identified as the most-popular sources of support followed by professional or formal sources of support such as discussions with supervisors or the use of counselors (Figure 7).

Finally, respondents to Surveys 2 and 3 were asked to identify the top sources of support they would like to see established by their employer. Mental health supports were the most-popular response for both surveys. These supports included improved access, coverage, and quality of mental health support and increased resources for staff wellness including mindfulness sessions, yoga, gym time, a place to relax, and opportunities to socialize.

In Survey 2, improving communications was identified as the second-most-popular recommendation, which included suggestions such as creating a safe place for discussions about pandemic-related challenges, and providing consistent, clear, unbiased, transparent, and personalized communications done at regular, timely intervals and scheduled effectively to allow time for planning. The second-most-popular theme for Survey 2 was directed at leadership (being receptive, open to listening, interested, present, responsive, supportive, interactive, able to engage with staff, and providing acknowledgement and recognition for staff). This theme was followed by recommendations related to improving workload management (e.g., permitting flexible schedules and work locations, improving staffing levels overall, accommodating leaves and sick time) and increasing financial compensation for all employees, including administrative staff and management, as well as introducing paid wellness days. As a Survey 2 participant explained “*It has been difficult to rest/rejuvenate. A few personal/paid days off would be helpful. The full-time grind is more arduous than normal, for a year now. Increased fatigue/stress/mentally & emotionally exhausted*”.

Interestingly, by Survey 3 the relative predominance of these categories had changed. While improving access to, coverage of, and quality of mental health support remained as the most-popular support suggested to be implemented, workload and financial compensation emerged as the second-most popular, while improving communications and leadership was lowered to third place.

## 4. Discussion

These findings offer a snapshot into the moral distress experience of BC HCWs at several time points during the COVID-19 pandemic. The longitudinal and regional aspects of this study improve our understanding of how moral distress experiences during COVID-19 manifest differently in different contexts and how they evolve over time in response to a continued stressor.

The majority of participants who self-selected to complete these surveys stated that they experienced moral distress, which is unsurprising given that they may have been attracted by the topic of this study and the title of the surveys, and, therefore, decided to participate due to their current situation at work.

The themes identified by the first survey offer an overview of participants’ common concerns during the initial stage of the public health response to the COVID-19 pandemic in BC. This initial stage was characterized by the presence of heightened uncertainty and the introduction of several restrictive social measures and pandemic management protocols across the healthcare system.

In this context, BC HCWs participating in this study stated that they experienced moral distress due to two main reasons: the impact that introduced changes were having on their capacity to serve patients and the new risks related to COVID-19 transmission and infection that they had to personally face. More specifically, BC HCWs were concerned about not being able to provide patient-centred, compassionate care, not being able to carry on their professional duties effectively, and about the impact of telehealth. They were also concerned over the risks that they, their colleagues, family members and friends were facing, including the presence of personally challenging circumstances in some cases. An additional source of moral distress centred on having disagreements with the pandemic management protocols that were being introduced.

These results align with previous studies [14,16,17,20] and highlight how by challenging standard professional routines and approaches to effective, compassionate, and patient-centred healthcare delivery, the implementation of pandemic-management protocols contributes to moral distress. They also suggest that the pressure to prioritize the health and safety of patients and communities over their own safety and that of those closest to them leaves HCWs feeling vulnerable and overburdened.

These sources of moral distress continued to be present during Surveys 2 and 3, despite the fact that those surveys were deployed at a time when some social restrictions had been lifted and progress was being made in the provincial vaccination program. This finding highlights the constant pressure that BC HCWs experienced during at least the first 15 months of the BC pandemic response and contrasts with that of Song [27]. In their study, which also included several surveys deployed at different times during the COVID-19 pandemic, the authors found that by stage 2 (24 October to 30 November 2020) the participants expressed “*resignation around adapting to the new normal*” [26] (p. 3). The fact that this theme did not emerge in our study highlights how context-specific the impact of the COVID-19 pandemic can be.

Importantly, participants identified two sources of current or anticipated ethical challenges: COVID-19 fatigue and the collateral impacts of the pandemic response. As the pandemic progressed, and HCWs had to continue to endure the moral stressor as they simultaneously experienced increasing fatigue. Interestingly, this fatigue was associated to an increased concern over the unanticipated consequences that the pandemic was causing to more vulnerable populations, with concerns over the quality of the clinical care provided and to disagreements with the protocols in place.

Study participants stated that they were relying on personal sources of support to cope with moral distress. This result highlights the importance of individual factors in managing this type of negative experiences, which are deeply personal [28]. However, as previous studies have indicated [29,30], broader institutional strategies are also required. Our study shows that it is important for HCWs that such institutional strategies are individualized; centred on meaningful, effective communications between leaders and staff members; and address operational concerns by, for example, managing workloads effectively and introducing financial compensation.

## 5. Limitations of the Research

This research was limited by several factors. First, the research was conducted solely in BC, which, compared to other areas of Canada and the world, had a unique experience of the pandemic in terms of the timing of certain events, including the impact of different waves of the pandemic, vaccine roll out, vaccine acceptance, etc. However, while the experience was unique, there were also many commonalities to other geographic regions, including significant disruptions to societal functioning due to public health measures such as lockdowns, social distancing, and travel restrictions as well as significant disruptions to the healthcare system. As such, although our study was geographically circumscribed, its results can likely apply across other jurisdictions.

A second limitation relates to the characteristics of the HCWs who self-selected to complete the survey. The majority of participants worked for two of the five health authorities, and the sample size is insufficient to be scientifically representative. Therefore, the results, discussions, and conclusions described in this paper are strictly related to the sample researched and are not necessarily representative of the experience of all HCWs in BC. However, as aspects of the pandemic response were unified across the province, results are, nonetheless, informative.

In addition, survey respondents were more likely to self-select if they were interested in the study, due to their experience of moral distress during the pandemic. However, because the aim of the study was to characterize the experience, impact, and response to moral distress during the pandemic, the self-selection bias likely had a positive impact on the permeation of moral distress in the sample.

Finally, the study was predominantly completed by individuals who identified as White females between the ages of 31 and 60. This demographic characteristic is reflective of the make-up of the healthcare system yet under-represents the voices of those who likely faced significant and unique impacts of the pandemic, including those who were non-White, newer immigrants, and of lower socio-economic status.

## 6. Future Research Directions

Several future research directions are suggested to improve the ability of the healthcare system to respond effectively to moral distress. In particular, our team plans to conduct additional work to test the reliability of the survey tool and complete follow-up research regarding the experience of healthcare workers who are racialized and face systemic barriers and inequities, in order to determine whether the coping mechanisms identified in this study are applicable, accessible, and likely to be effective to them and the communities they represent. In addition, further efforts to address psychological health and wellness in effective, low-barrier and culturally appropriate manners are essential. Finally, further work and consideration should be given to how to prepare healthcare workers during early stages of their careers for the conflicting values and responsibilities they may face during public-health emergencies.

## 7. Conclusions

This qualitative study showed that many BC HCW survey participants experienced moral distress during the initial stages of the COVID-19 pandemic as they struggled to provide effective, compassionate, and patient-centred care, while also facing significant personal risks. Many also disagreed with aspects of the pandemic management protocols. Results demonstrate that COVID-19 fatigue and the collateral impact of the pandemic introduce additional layers to HCWs’ experiences of moral distress. Coping strategies were identified at the individual, team, and organizational levels, including: providing personalized support; increasing the effectiveness of the communications between leaders and staff members; addressing operational concerns by managing workloads effectively; and introducing financial compensation. These strategies can be used by organizations as potential starting points to facilitate both individual and system recovery.

This study adds to the literature on moral distress by highlighting the scale of the impact that pandemics can have on all aspects of the healthcare system, that is, beyond critical care, which is the main focus of the moral-distress literature. It also highlights how the societal impact can be a source of moral distress for HCWs. Finally, study results identify specific measures that healthcare organizations can implement to mitigate the experience of moral distress and inform healthcare leaders about the importance of maintaining and retaining a skilled workforce that has been significantly battered by the pandemic. This is particularly important given the impacts on healthcare workers and the healthcare system by long COVID-19 and the continuing impacts of variants of concern, leading to staffing shortages as well as supply-chain shortages. These shortages continue to place pressure on the healthcare system in multiple ways, including complex and morally distressing triage decisions and fatigue. Ongoing monitoring of the impacts of long COVID-19 and the pandemic’s successive waves on the moral wellness of staff are essential to ensure adaptive and evolving strategies to aid in healthcare workers’ wellbeing and overall system function.

## Figures and Tables

**Table 1:** Demographics of survey participants ^1^.

	Survey 1 8 May 2020–28 May 2020	Survey 2 22 October 2020–17 March 2021	Survey 3 18 March 2021–31 July 2021
Completed surveys	N = 135	N = 320	N = 145
Age			
Under 20	0%	1%	0%
21–30	10%	16%	12%
31–40	31%	25%	27%
41–50	24%	25%	23%
51–60	25%	23%	23%
61–70	6%	8%	12%
71–90	1%	0%	1%
No response	2%	1%	1%
Gender			
Female	78%	82%	80%
Male	19%	13%	15%
Non-binary or Other	0%	0.3%	3%
No response	4%	4%	2%
Race or Ethnicity			
White	80%	75%	70%
Indigenous (Canadian)	2%	3%	2%
Indigenous (non-Canadian)	NA ^2^	0.3%	1%
South Asian	4%	6%	4%
Chinese	3%	6%	11%
Black	0%	0.3%	1%
Filipino	2%	2%	5%
Latin American	2%	1%	3%
Arab	0%	0.3%	1%
Southeast Asian	0%	1%	5%
West Asian	1%	1%	1%
Korean	1%	0%	1%
Japanese	0%	1%	2%
Other	2%	3%	5%
Prefer not to respond	5%	6%	3%

^1^ Please note that numbers may not equal 100% due to rounding and, for race/ethnicity, because participants could select more than one option. ^2^ Indigenous (non-Canadian) was not offered as an option in Survey 1.

**Table 2:** Participants’ perspectives of the main ethical challenges faced, or to be faced, by HCW during the pandemic.

Ethical Challenges	Survey 1 (*N* = 248)	Survey 2 Completed Surveys (*N* = 418)	Survey 3 Completed Surveys (*N* = 220)
COVID-19-fatigue: being tired of all COVID-19-related matters	*N* = 0	*N* = 271	*N* = 119
Collateral impact of COVID-19, e.g., exposing social inequities in healthcare, affecting population’s mental health	*N* = 16	*N* = 205	*N* = 82
Clinical services have been changed in ways that prevent healthcare workers from providing family-centred, compassionate patient care	*N* = 47	*N* = 170	*N* = 73
Being required to work regardless of the risk to their family members or colleagues	*N* = 9	*N* = 165	*N* = 64
Being required to work regardless of the personal risk	*N* = 20	*N* = 162	*N* = 72
Disagreements with colleagues or leadership regarding the COVID-19 protocols that should be implemented	*N* = 32	*N* = 130	*N* = 46
Lack of a safe environment to discuss disagreements with colleagues or leadership regarding the COVID-19 protocols that should be implemented	*N* = 13	*N* = 99	*N* = 41
COVID-19 safety protocols prevent healthcare workers from carrying out their professional duties	*N* = 11	*N* = 74	*N* = 21
Effectiveness of tele-health	*N* = 8	*N* = 33	*N* = 16

## Data Availability

The de-identified aggregated study findings are contained within this article. Individual survey data are available on request from the corresponding author and will be de-identified prior to sharing.

## Supplementary Materials

The following supporting information can be downloaded at: https://www.mdpi.com/article/10.3390/ijerph19159701/s1, Supplementary S1: Survey 1; Supplementary S2: Surveys 2 and 3.
