# Supplementary material for: COVID-19 Outbreak: Understanding Moral-Distress Experiences Faced by Healthcare Workers in British Columbia, Canada
Source: Int J Environ Res Public Health. 2022 Aug 6;19(15):9701. doi: 10.3390/ijerph19159701 (PMC9368033; doi:10.3390/ijerph19159701)
Supplement: Supplementary file 1 [file ijerph-19-09701-s001.zip › Supplementary Material S1.pdf]

SUPPLEMENTARY MATERIALS - S1

Survey 1

Q 1 of 12: Have you taken this survey before?

Yes
No

Q 2 of 12: How old are you?

Under 20
21-30
31-40
41-50
51-60
61-70
71-80
81-90
Prefer not to respond

Q 3 of 12: What gender do you identify with?

Female
Male
Non-binary
Other
Prefer not to respond

Q 4 of 12: What is your present religious affiliation, if any?

Roman Catholicism
Protestantism
Anglicanism
Islam
Hinduism
Sikhism
Buddhism
Judaism
Non-Religious
Other
Prefer not to respond
Please, specify "Other"

Q 5 of 12: What race or ethnicity do you identify with? (Select all that applies).

White

Indigenous (First Nations, Inuit, Metis)
Indigenous (non-Canadian)
South Asian (e.g., East Indian, Pakistani, Sri Lankan, etc.)
Chinese
Black
Filipino
Latin American
Arab
Southeast Asian (e.g., Vietnamese, Cambodian, Laotian, Thai, etc.)
West Asian (e.g., Iranian, Afghan, etc.)
Korean
Japanese
Other
Prefer not to respond

Q 6 of 12: What Health Authority are you part of?

Fraser Health Authority (FHA)
Interior Health Authority (IHA)
Northern Health Authority (NHA)
Vancouver Island Health Authority (VIHA)
Vancouver Coastal Health Authority (VCHA)
Provincial Health Services Authority (PHSA)
Providence Healthcare (PHC)

Q 7 of 13 Have you experienced moral distress in your practice during the COVID-19 outbreak?

Yes
No

If yes, please describe those experiences. ________________________________

____

Q 8 of 13 What type of impact are those experiences having on you professionally? Personally?

____

Q9 of 13 What do you think are the main factors that are currently leading to moral distress?

____

Q 10 of 13 What resources are available to you right now to help you deal with moral distress?

____

Q 11 of 13 Do you anticipate that sources of moral distress will emerge as the situation evolves?

Yes
No

If yes, please describe those future sources of moral distress.

____

Q12 of 13 What resources to help dealing with moral distress do you think might be helpful and should be implemented or developed? ____

Q 13 of 13 Generally speaking, what are the top three ethics issues you've encountered (maybe not directly experienced by you but by your colleagues) during the pandemic?
