# Supplementary material for: COVID-19 Outbreak: Understanding Moral-Distress Experiences Faced by Healthcare Workers in British Columbia, Canada
Source: Int J Environ Res Public Health. 2022 Aug 6;19(15):9701. doi: 10.3390/ijerph19159701 (PMC9368033; doi:10.3390/ijerph19159701)
Supplement: Supplementary file 1 [file ijerph-19-09701-s001.zip › Supplementary Material S2.pdf]

SUPPLEMENTARY MATERIALS - S2

Surveys 2 and 3

Q 1 of 12: Have you taken this survey before?

Yes
No

Q 2 of 12: How old are you?

Under 20
21-30
31-40
41-50
51-60
61-70
71-80
81-90
Prefer not to respond

Q 3 of 12: What gender do you identify with?

Female
Male
Non-binary
Other
Prefer not to respond

Q 4 of 12: What is your present religious affiliation, if any?

Roman Catholicism
Protestantism
Anglicanism
Islam
Hinduism
Sikhism
Buddhism
Judaism
Non-Religious
Other
Prefer not to respond

- Please, specify "Other"

Q 5 of 12: What race or ethnicity do you identify with? (Select all that applies).

White

Indigenous (First Nations, Inuit, Metis)
Indigenous (non-Canadian)
South Asian (e.g., East Indian, Pakistani, Sri Lankan, etc.)
Chinese
Black
Filipino
Latin American
Arab
Southeast Asian (e.g., Vietnamese, Cambodian, Laotian, Thai, etc.)
West Asian (e.g., Iranian, Afghan, etc.)
Korean
Japanese
Other
Prefer not to respond

Q 6 of 12: What Health Authority are you part of?

Fraser Health Authority (FHA)
Interior Health Authority (IHA)
Northern Health Authority (NHA)
Vancouver Island Health Authority (VIHA)
Vancouver Coastal Health Authority (VCHA)
Provincial Health Services Authority (PHSA)
Providence Healthcare (PHC)

Q 7 of 12: Have you experienced moral distress in your practice during the COVID-19 outbreak? (Moral distress is something that can happen when you know the ethically correct action that needs to take place but you feel powerless to take that action due to several reasons such as fear, poor communication, pressure from others, or institutional policies or procedures.)

Yes
No

Q 7.1 of 12: If yes, please, select all that apply.

I have been required to work regardless of the risk to myself
I have being required to work regardless of the risk to my family members or colleagues
I have being required to work regardless of my personal challenging circumstances
The pandemic management protocols put in place prevent me from carrying out my professional duties effectively
Clinical services have been modified compromising my ability to provide family-centered, compassionate clinical care
I disagree with the COVID-19 safety protocols that had been implemented in my work place
I am concerned that tele-health compromises my ability to provide optimal clinical care

I am concerned that tele-health has significant privacy and security risks
Other
- Please, specify "Other".
- Please, use this box to enter any additional explanation to your answer.

Q 7.2 of 12: What type of impact are those experiences having on you professionally and personally?

Stress
Anxiety
Feeling helpless
Increased empathy
Decreased empathy
Irritability
Difficulties sleeping
Other

Q 8 of 12: In your opinion, what are the main ethical challenges that healthcare employees are currently facing or will be facing in response to the COVID-19 pandemic?

Being required to work regardless of the personal risk
Being required to work regardless of the risk to their family members or colleagues
Clinical services have been changed in ways that prevent healthcare workers from providing family-centered compassionate patient care
COVID-19 safety protocols prevent healthcare workers from carrying out their professional duties
Effectiveness and security of tele-health
Disagreements with colleagues or leadership regarding the COVID-19 protocols that should be implemented
Lack of a safe environment to discuss disagreements with colleagues or leadership regarding the COVID-19 protocols that should be implemented
Collateral impact of COVID-19 , e.g. exposing social inequities in healthcare, affecting population's mental health
COVID-19-fatigue: being tired of all COVID-19 related matter
Other

Q 9 of 12: What are the main sources of support you have used or are using to cope with the negative psychological impact of COVID-19 ?

Formal opportunities to discuss the issues with supervisors, leaders and colleagues
Informal support provided by colleagues, family members or friends
Psychologist / Counsellor / Other mental health professionals
Written communications provided by my employer
Self-care resources (mindfulness, online yoga etc.)
Other

Q 10 of 12: How would you rate the following institutional sources of support provided during the COVID-19 pandemic outbreak?

Providing formal opportunities to discuss the issues with supervisors , leaders and colleagues (leave blank if not applicable to you).

Providing access to mental health specialists such as psychologists, counsellors, peer-groups (leave blank if not applicable to you).

Providing clear and timely written communications via email (leave blank if not applicable to you). (Place a mark on the scale above)

Q 11 of 12: What are the top three sources or support you would like to see put in place by your employer?

Source of Support #1:
Source of Support #2:
Source of Support #3:

Q 12 of 12: What is your work role? (Please do not include your actual title or any information that could be used to identify you).

Nurse
Physician
Paramedic
Allied Health Professional
Researcher
Administrative Personnel
Manager
Executive
Other
Prefer not to respond
